# Thermodynamics, Environmental and Sustainability Impacts of a Turbofan Engine Under Different Design Conditions Considering Variable Needs in the Aviation Industry

**DOI:** 10.1002/gch2.202300205

**Published:** 2024-01-18

**Authors:** Hakan Aygun, Mohammad Rauf Sheikhi, Hakan Caliskan

**Affiliations:** ^1^ Department of Aircraft Airframe and Power‐Plant Firat University Elazig 23119 Türkiye; ^2^ The State Key Laboratory of Heavy‐duty and Express High‐power Electric Locomotive Central South University Changsha 410075 China; ^3^ Key Laboratory of Traffic Safety on Track of Ministry of Education School of Traffic & Transportation Engineering Central South University Changsha Hunan 410075 China; ^4^ National & Local Joint Engineering Research Center of Safety Technology for Rail Vehicle Central South University Changsha 410075 China; ^5^ Department of Mechanical Engineering Faculty of Engineering and Natural Sciences Usak University Usak 64200 Türkiye

**Keywords:** entropy, environmental effect, exergy, sustainability, turbofan

## Abstract

In this study, thermodynamic analysis is implemented to the kerosene‐fuelled high by‐pass turbofan (HBP‐TF) engine to assess entropy, exergy, environmental, and sustainability metrics for different design variables such as pressure ratio of high‐pressure compressor (HPC‐PR) ranging from 7.5 to 8.5 and turbine inlet temperature (TIT) varying from 1400 to 1525 K considering variable needs in the aviation industry. As a novelty, entropic improvement potential (EIP) index for turbomachinery components and specific irreversibility production for the whole engine are calculated. Sustainability‐based parameters for different cases are compared with the baseline values of the HBP‐TF engine. The combustor has the highest entropy production of 44.4425 kW K^−1^ at the baseline. The higher TIT increases the entropy production of the combustor by 16.56%, whereas the higher HPC‐PR decreases it by 5.83%. The higher TIT and HPC‐PR favorably affect the sustainable efficiency factor of the engine, which is observed as 1.5482 at baseline and increases by 4.5% and 0.058% with the increment of TIT and HPC‐PR, respectively. The higher TIT and higher HPC‐PR results in lowering sustainability of the engine. The specific irreversibility production of the engine decreases by 3.78% and 0.1171% respectively, as TIT and HPC‐PR reach the highest point considered in the study.

## Graphical abstract

Thermodynamic analysis is applied to the kerosene fuelled high by‐pass turbofan (HBP‐TF) engine to assess entropy, exergy, environmental and sustainability metrics for different design variables. As a novelty, entropic improvement potential index for turbomachinery components and specific irreversibility production for the whole engine are computed. Sustainability‐based parameters for different cases are compared with the baseline values of HBP‐TF engine.

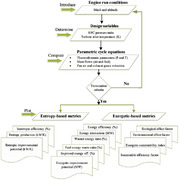

## 1 Introduction

The aviation industry has been growing rapidly in recent years, and despite the advancement in aircraft performance, the industry's effect on climate change is a major concern due to fuel combustion.^[^ ^1^ ^]^ With the increasing use of gas turbine engines in civil aircraft, the fuel emissions of pollutants from these engines have also increased sharply. Moreover, the usage of fossil fuels in transportation has faced significant challenges due to getting related legislation more strict. To decrease the dependency on fossil fuels, 30–40% of the thermal efficiency of gas turbines is required to increase with novel technologies such as solid oxide fuel cell gas turbines.^[^ ^2^ ^]^ According to the International Energy Agency, aviation is probably the most difficult transport sector to decarbonize, due to its cost and size.^[^ ^3^ ^]^ Groundbreaking technology is much‐needed, as, in the 2010s, flying grew to become more popular than ever. By 2019, the number of passengers reached a staggering 4.6 billion, with the number expected to double by 2037.^[^ ^4^ ^]^ Despite the total quantity of emissions from aviation is less than land‐based vehicles, their impact on global warming is great as cruise flights are between the upper tropopause and lower stratopause. Therefore, many studies have focused on the reduction of aircraft emissions, especially in the last three decades.^[^ ^5^ ^]^

Aircraft engines are the most significant component of flight operations. Each phase of flight requires different power demands from the aircraft engines. The efficiency, economy, sustainability, and environmental implications of the engines, which are the aircraft's energy conversion system, should be evaluated to describe the performance characteristics of the operations. Thermodynamic analysis is a promising methodology that covers all these analytical procedures and has several assessment indicators.^[^ ^6^, ^7^ ^]^ The exergy, a measure of thermodynamic quality or capability, has been proposed by several researchers as a basis for indicators of the environmental effects generated by resource extraction and waste emissions linked with human activities. Exergy‐based indicators are thought to be more comprehensive and informative than other metrics for assessing and comparing environmental effects since these need fewer assumptions and subjective decisions.^[^ ^8^, ^9^ ^]^ A system with a lower rate of waste exergy is more useful and can perform more work. A less efficient system has a higher rate of waste exergy due to the destruction of exergy and therefore lower potential for working.^[^ ^10^ ^]^ The exergy efficiency, which is one of the most significant indicators for an engine's sustainability, is primarily determined by the exergy input and their required output. At all stages of a flight, the input‐output exergetic values of each engine component have a significant impact on the turbofan engine's exergy efficiency.^[^ ^11^ ^]^

Exergy analysis is a technique used to design more efficient power plants. In power plant applications, the main aim of exergy analysis is to identify and reduce irreversibility in each component of the plant. Many scholars have used the concept of exergy in a wide range of industrial operations to date. Another appealing feature of exergy analysis is that it is a useful tool for achieving the construction of energy‐efficient systems with tiny environmental footprints. In this framework, an open gas turbine system was subjected to an exergy study by Morosuk et al.^[^ ^12^ ^]^ They highlighted the significance of evaluating a system component's thermodynamic performance to establish which component was causing higher exergy destructions. The relationship between exergy destruction, exergo‐economic, exergo‐environmental, and sustainable development was investigated by Dincer and Rosen.^[^ ^13^ ^]^ They used exergy destruction as a tool for economic planning, resource optimization, and global, and reducing environmental pollution at different scales in their work.

Sohret et al.^[^ ^14^ ^]^ investigated the exergy of a turbofan engine for an unmanned aerial vehicle. The engine was a Rolls‐Royce AE3007H high bypass turbofan engine specially built for the Global Hawk. Within the engine, the combustion chamber and afterburner were determined to have the maximum exergy destruction. Furthermore, Balli et al.^[^ ^15^ ^]^ performed energetic and exergetic assessments on a turboprop aircraft with four engine loads: 75%, 100%, military mode, and takeoff mode. The mechanical shaft power and the kinetic exergy rate of the exhaust gas were used to calculate the efficiencies. The takeoff mode of a turboprop was found to have the greatest energetic and exergetic efficiency rates, but the engine's combustion chamber had the highest irreversibility. Caglayan et al.^[^ ^16^ ^]^ reported the findings of advanced exergy assessments performed at various environment temperatures on a ceramic tile plant's cogeneration system. The exergy destruction was maximum in the combustion chamber, while the exergy efficiency was lowest in the wall tile dryer (17.43% at 30 °C) and ground tile dryer (23.13% at 30 °C). They also observed that, while the endogenous exergy destruction value of the cogeneration system was greater than the external exergy destruction value, the connections between the components appeared weak. Additionally, exergy and life cycle‐based environmental and enviro‐economic studies on a micro‐gas turbine that operated with natural gas and alternate mixes of natural gas‐ammonia and methanol were investigated by Ayaz et al.^[^ ^17^ ^]^ The exergy destruction was observed to occur primarily in the burner, and both supplemental fuels minimized exergy destruction in the burner, exhaust gas heat exchanger and air preheater. They also discovered that adding extra fuel lowers the system's exergy efficiency. Moreover, Chaudhary et al.^[^ ^18^ ^]^ proposed a concept of hybridizing air‐blade cooled turboprop engines with SOFC system, which can utilize the waste heat of the engine exhaust and increase the overall performance of the hybrid system. They performed a thermodynamic analysis of the turboprop‐SOFC system based on the 1st and 2nd laws of thermodynamics and investigated the effects of various parameters such as compressor pressure ratio, turbine inlet temperature, and airflow rate on the system. They evaluated the energy and exergy losses in each component of the hybrid system and found that the integration of the SOFC with the turboprop engine can increase the efficiency by ≈12–13% and reduce the exergy degradation in the fuel cell. On the other hand, Ekrataleshian et al.^[^ ^19^ ^]^ performed energy, exergy, and exergoeconomic evaluations on the components of a turbojet engine. According to their findings, the combustion chamber has the largest exergy destruction and the biggest improvement potential. The greatest exergy efficiency among the system components belongs to an air compressor, and they discovered that increasing the turbine inlet temperature enhances exergy destruction and energy efficiency of turbojet engines. Ahmadi et al.^[^ ^20^, ^21^ ^]^ investigated a thermodynamic study of an irreversible Brayton cycle with the goal of optimizing the Brayton cycle performance using the multi‐objective optimization algorithm scenarios and they mentioned that the multi‐objective algorithm is an acceptable method for optimizing the Brayton cycle‐based system performance. Chaudhary et al.^[^ ^22^ ^]^ developed a thermodynamic model of a hybrid cycle combining a solid oxide fuel cell (SOFC) with an intercooled‐recuperated gas turbine (ICGT). They analyzed the performance of the hybrid cycle based on the first and second laws of thermodynamics and optimized it by minimizing the entropy generation. They reported that the hybrid cycle could achieve an optimal efficiency of 74.13% and provided a performance map for the system. A modified stability index and exergy indices have been proposed to compare different gas turbine cycles, including a hybrid configuration with a SOFC, by Sinha et al.^[^ ^23^ ^]^ They used MATLAB for energy‐exergy modeling and stability analysis of the system and its components. They found that the IC‐SOFC‐RGT hybrid cycle has the highest efficiency and stability index among the three configurations and identified the most components in each configuration.

In previous studies, the exergetic efficiency and sustainability‐based metrics were used for the different conditions of aero‐engines based on various bypass modes, turbine inlet temperatures, flight phases, dynamic loads, and results of the exergo‐sustainability analysis, which can be useful tools for designers.^[^ ^24^, ^25^, ^26^ ^]^ Thus, it is important to do research that demonstrates the possible advantages that can be obtained in exergetic and sustainability‐based metrics for a turbofan engine. In this study, the analyses of thermodynamics involving the first and second laws are conducted for the high by‐pass turbofan (HBP‐TF) engine under different cases. As a difference from previous studies, the entropic improvement potential (EIP) metric and specific irreversibility production (SIP) are measured for the HBP‐TF engine for the first time. The main goal of the study is to observe the discrepancies in thermodynamic metrics from the baseline for different cases. Namely, it is analyzed how the variation of TIT and HPC‐PR affects the potential savings in terms of sustainability of the engine. According to the literature survey, no studies have focused on entropy, exergy, and environmental and sustainability metrics together for several design points. The main contributions of this paper to the literature can be expressed as follows:

- Observing the effects of TIT and HPC‐PR on entropy parameters such as entropy production for the HBP‐TF engine in different cases.
- Analyzing the responses of exergetic parameters such as exergy efficiency, wasted exergy ratio, fuel exergy waste ratio, exergetic improvement potential, and improved exergy efficiency to different TIT and HPC PR regarding for the engine.
- Estimating environmental and sustainability indicators such as ecologic effect factor, environmental effect factor, exergetic sustainability index, and sustainable efficiency factor for different TIT and HPC PR values.
- Assessing TIT and HPC PR metrics for the overall engine parameters, especially environmental and sustainability indicators, by comparing their baseline values.

## 2 System Description

**Figure 1:**
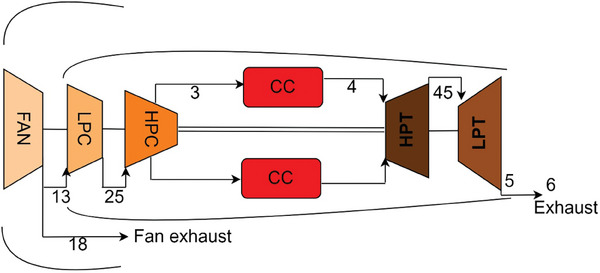
Schematic demonstration of the HBP‐TF engine.^[^ ^28^ ^]^

The kerosene‐fueled high bypass turbofan engine is studied as a system in which the primary flow is directed to the combustor, whereas the secondary flow is sent exclusively to the Fan. The bypass duct receives 85% of the engine's airflow, which is accelerated by the Fan and produces 80% of the engine's thrust. CFM56‐7B engines are commonly used in the Boeing 737 (B‐737) commercial aircraft series, as well as the AEW&C and B‐8 Poseidon military aircraft.^[^ ^27^, ^28^ ^]^ Over 8000 CFM56‐7Bs are used in the B‐737 series. With a 5.1 bypass ratio and a 32.8 total pressure ratio, the thrust produced is 121 kN. The engine comprises a single‐stage fan, a 12‐stage compressor that includes a 3‐stage low‐pressure compressor (LPC) and a 9‐stage high‐pressure compressor (HPC), an annular combustion chamber, and a 5‐stage turbine that includes a 1‐stage high‐pressure turbine (HPT) and four stages low‐pressure turbine (LPT).^[^ ^29^ ^]^ **Figure** **1** presents a typical HBP‐TF engine representing the CFM56‐7B engine. On the other hand, this engine is subjected to thermodynamic analysis by varying design variables for different cases. It could be noteworthy that the corresponding turbine inlet temperature (TIT) and high‐pressure compressor pressure ratio (HPC‐PR) values for the considered engine are 1450 of TIT and 8.1 of HPC‐PR.

## 3 Methodology

### 3.1 Entropy Relations

The entropy term is associated with the second law of thermodynamics. It is a property of the substance and is transferred to both mass flow and heat transfer.^[^ ^31^ ^]^ The second law could be expressed with entropy balance. When all actual processes take place, entropy production in the system occurs. Otherwise, this process could not be completed. In other words, entropy production originates from the microscopic disorder of the molecules.^[^ ^31^ ^]^ The entropy changes equal to the entropy production and the difference between input and output entropies which can be expressed as follows^[^ ^29^ ^]^:

(1) $$S_{in}-S_{out}+S_{gen}=\Delta S_{system}$$

(2) $$\frac{dS_{CV}}{dt}=\sum \frac{Q_{J}}{T_{J}}+\sum m_{in}s_{in}-\sum m_{out}s_{out}+S_{gen}$$

(3) $$0=\sum \frac{Q_{J}}{T_{J}}+\sum m_{in}s_{in}-\sum m_{out}s_{out}+S_{gen}$$

where *S_in_* and *S_out_* represent input and output entropies, whereas *S_gen_* and Δ*S_system_* are entropy generation and the change of entropy, respectively. Moreover, *Q_J_* denotes heat transfer rate for non‐adiabatic components.

For the components that consume power, the isentropic efficiency is given in Equation 4 while for the components that produce power, this index is expressed in Equation 5.

(4) $$\eta_{Fan,LPC,HPC}=\frac{\Delta h_{t,isentropic}}{\Delta h_{t,actual}}$$

where η_*Fan*,*LPC*, *HPC*_ represents isentropic efficiency of Fan, LPC, and HPC whereas Δ*h* denotes the enthalpy difference

(5) $$\eta_{HPT,LPT}=\frac{\Delta h_{t,actual}}{\Delta h_{t,isentropic}}$$

Entropic improvement potential is first measured for turbomachinery components such as compressor and turbine units. This index means how much an improvement rate is obtained when removing irreversibility or entropy production that occurred in these components. It is expressed as:

(6) $$EIP_{RC}=\left(1-\eta_{isentropic,RC}\right).\left(S_{gen,RC}\right)$$

where *EIP_RC_* and *S*_*gen*,*RC*_ denote entropic improvement potential and entropy production for rotary components.

### 3.2 Exergy Relations

Exergy analysis may be used in any system that involves heat and work transfers in general. Exergetic efficiency depicts the system's efficiency related to its reference environment. Increasing the efficiency of energy and exergy conversion processes, and therefore reducing fuel usage, is a primary goal of lowering a system's environmental damage effect. Some exergy analysis parameters are used to do enhanced exergy analysis.

Exergy destruction is found by subtracting from fuel exergy to product exergy. It is shown as^[^ ^32^, ^33^ ^]^:

(7) $$\sum Ex_{F}-\sum Ex_{Pr}=\sum Ex_{Dest}$$

where Ex means exergy and the subscripts *F* and *Pr* denote “fuel” and “product”, respectively.

Exergy efficiency is the ratio of product exergy to fuel exergy. It is given as:

(8) $$\eta_{ex}=\frac{Ex_{Pr}}{Ex_{F}}$$

Waste exergy ratio (*WexR*) is the ratio of wasted exergy of the *k*th component to total wasted exergy. It is presented as follows^[^ ^32^ ^]^:

(9) $$WExR_{k}=\frac{Ex_{WE,k}}{Ex_{WE,engine}}=\frac{Ex_{D,k}+Ex_{L,k}}{Ex_{WE,engine}}$$

Fuel exergy waste ratio (*FexWR*) is wasted exergy of the *k*th component to fuel exergy. It is expressed as:

(10) $$FExWR_{k}=\frac{Ex_{WE,k}}{Ex_{F,engine}}=\frac{Ex_{D,k}+Ex_{L,k}}{Ex_{F,engine}}$$

The exergetic improvement potential (*ExIP*) is the relation between exergy efficiency and exergy destruction of the kth component.^[^ ^34^ ^]^ It is presented as:

(11) $$ExIP_{k}=\left(1-\eta_{ex}\right)Ex_{D,k}$$

Improved exergy efficiency is determined from *ExIP* and exergy efficiency as follows^[^ ^35^, ^36^ ^]^:

(12) $$\Psi =\frac{Ex_{Pr}}{Ex_{F}-ExIP}$$

### 3.3 Environmental & Sustainable Metrics

The ecological effect factor (*EcoEF*) is computed from exergy efficiency as follows^[^ ^30^ ^]^:

(13) $$EcoEF_{k}=\frac{Ex_{F}}{Ex_{Pr}}=\frac{1}{\eta_{ex,k}}$$

Environmental effect factor (*EEF*) is the ratio of “fuel exergy waste ratio” to “exergy efficiency”^[^ ^35^ ^]^:

(14) $$EEF_{k}=\frac{FExWR_{k}}{\eta_{ex,k}}$$

Exergetic sustainable index (*ExSI*) is found from the environmental effect factor as follows:

(15) $$ExSI_{k}=\frac{1}{EEF_{k}}$$

To find the sustainable efficiency factor (*SEF*), calculation of exergy efficiency is required. It is expressed as^[^ ^32^ ^]^:

(16) $$SEF_{k}=\frac{1}{1-\eta_{ex,k}}$$

Specific irreversibility production is the ratio of total exergy destruction to the net thrust of the engine as follows^[^ ^33^ ^]^:

(17) $$SIP_{engine}=\frac{\sum Ex_{Dest}}{\tau_{engine}}$$

where τ_*engine*_ represents net thrust of the engine.

**Figure 2:**
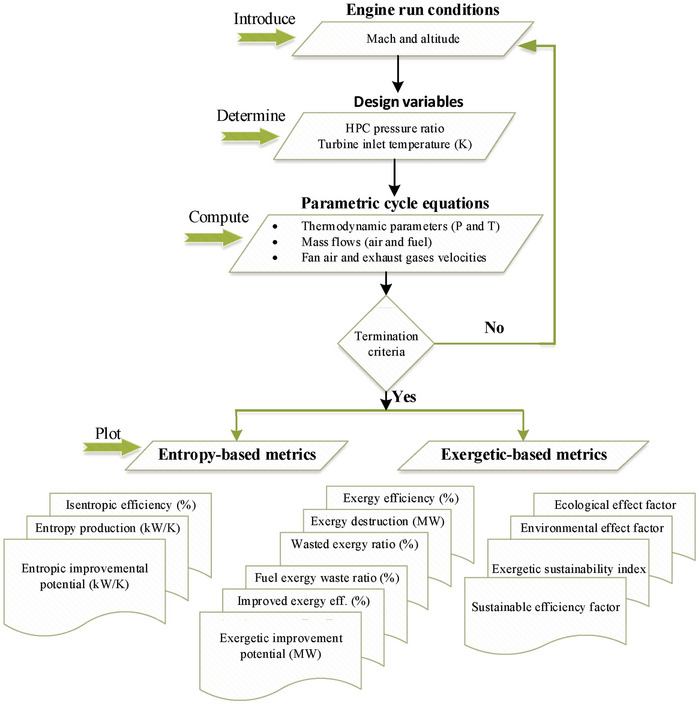
Flowchart of entropy and exergy analyses regarding HBP‐TF engine.

**Figure** **2** presents the analysis steps for obtaining entropy and exergy‐based metrics for each condition.

When performing the analyses, some conditions are assumed, which can be given as follow:

- Ambient pressure and temperature are 101 kPa and 288 K, respectively.
- A steady‐state condition is considered for the engine operation hold for all cases.
- The used fuel (C_12_H_23_) is kerosene, and its lower heating value is 42.8 MJ kg^−1^.
- The turbomachinery components such as compressor and turbine are in adiabatic condition.
- The combustion occurs at a stoichiometric air‐to‐fuel ratio.
- The potential and kinetic exergies are ignored.

## 4 Results and Discussion

**Figure 3:**
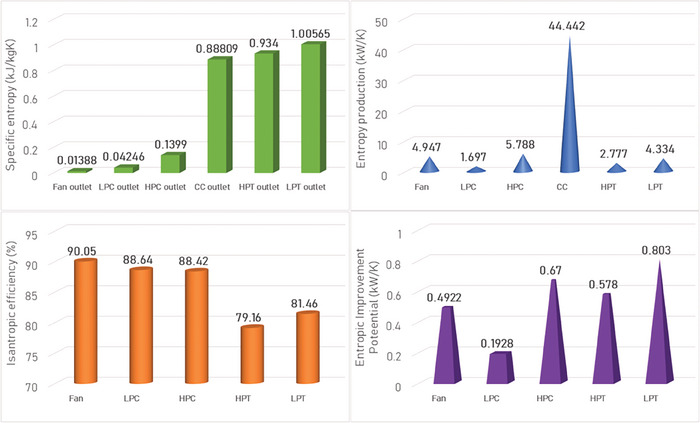
Entropy metrics regarding HBP‐TF engine at baseline.

**Figure 4:**
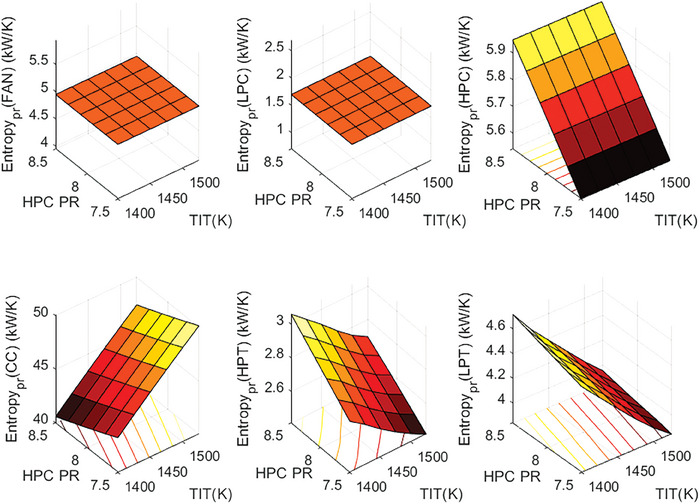
Entropy production of the HBP‐TF engine components at different cases.

**Figure 5:**
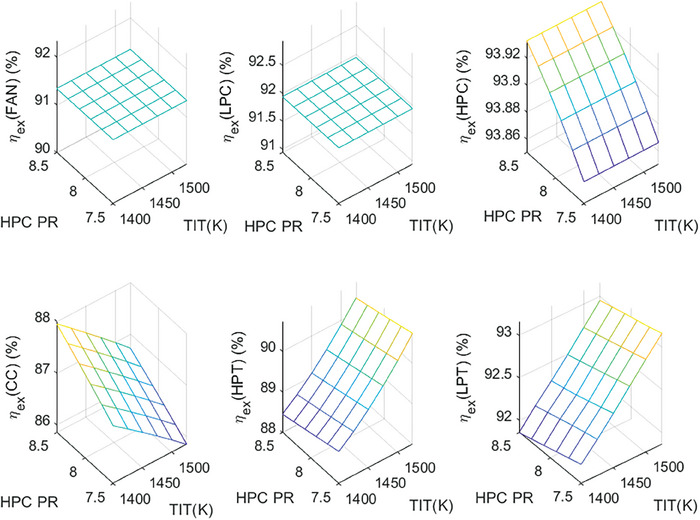
Exergy efficiency of the HBP‐TF engine components at different cases.

**Figure 6:**
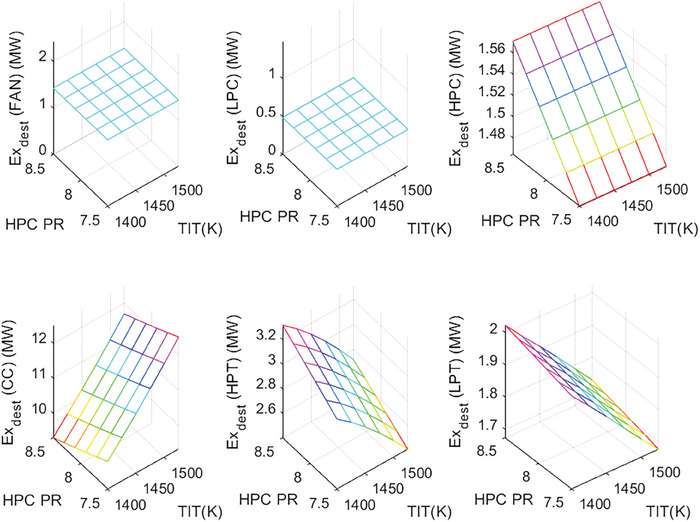
Exergy destruction of the HBP‐TF engine components at different cases.

**Figure 7:**
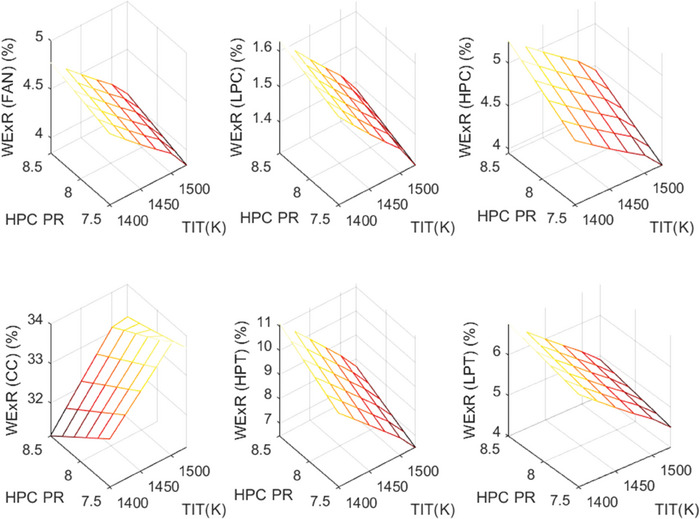
Wasted exergy ratio of the HBP‐TF engine components at different cases.

**Figure 8:**
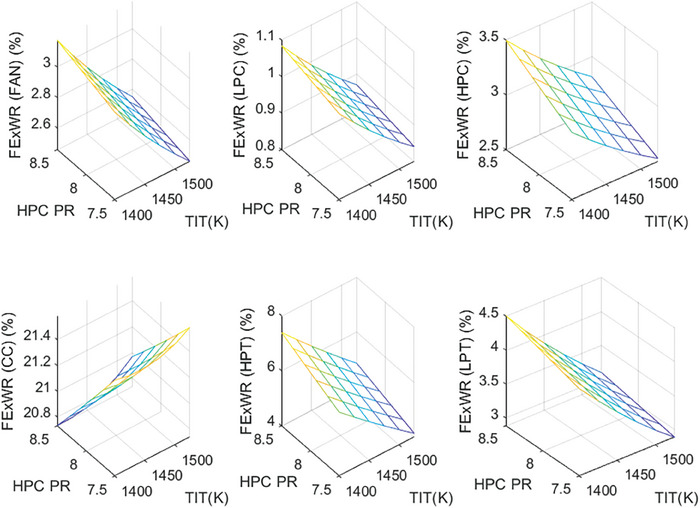
Fuel exergy waste ratio of the HBP‐TF engine components at different cases.

**Figure 9:**
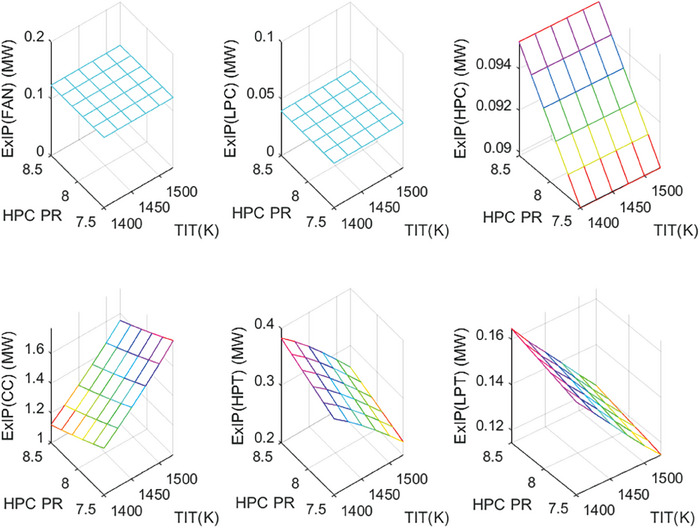
Exergetic improvement potential of the HBP‐TF engine components at different cases.

**Figure 10:**
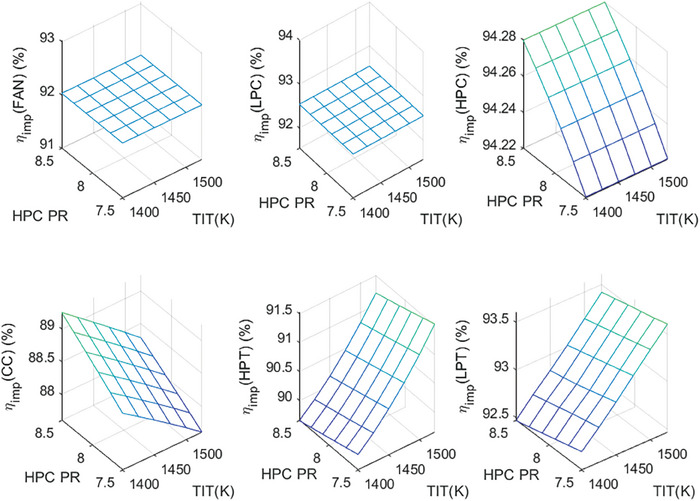
Improved exergy efficiency of the HBP‐TF engine components at different cases.

**Figure 11:**
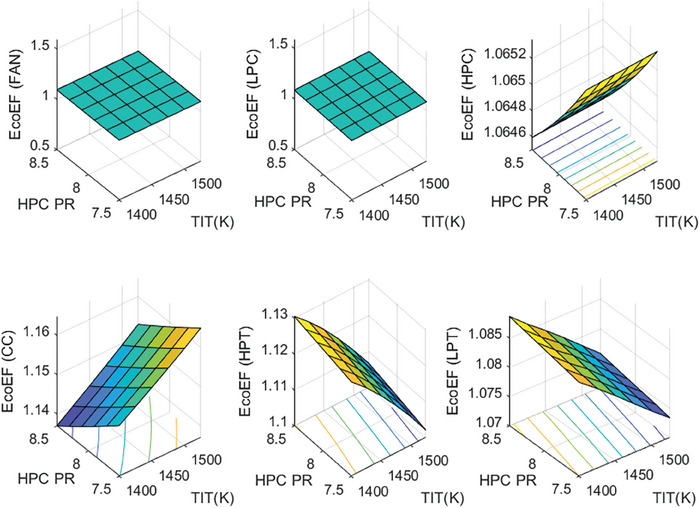
Ecological effect factor of the HBP‐TF engine components at different cases.

**Figure 12:**
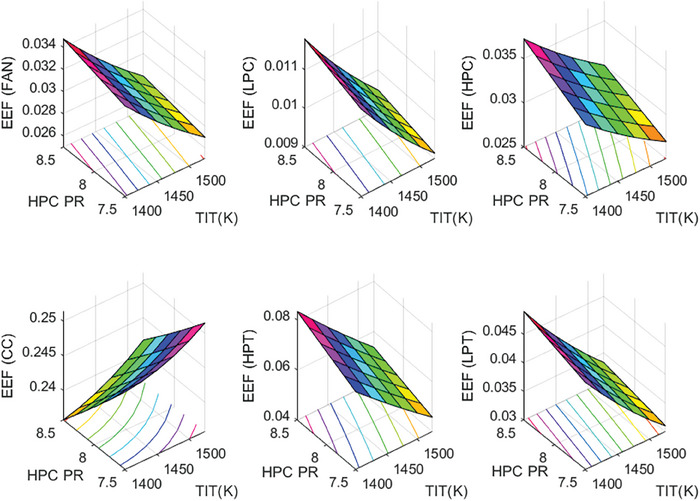
Environmental effect factor of the HBP‐TF engine components at different cases.

**Figure 13:**
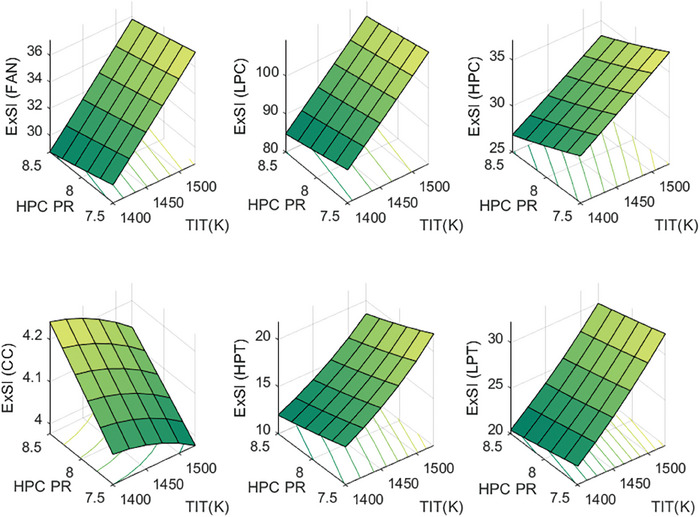
Exergetic sustainability index of the HBP‐TF engine components at different cases.

**Figure 14:**
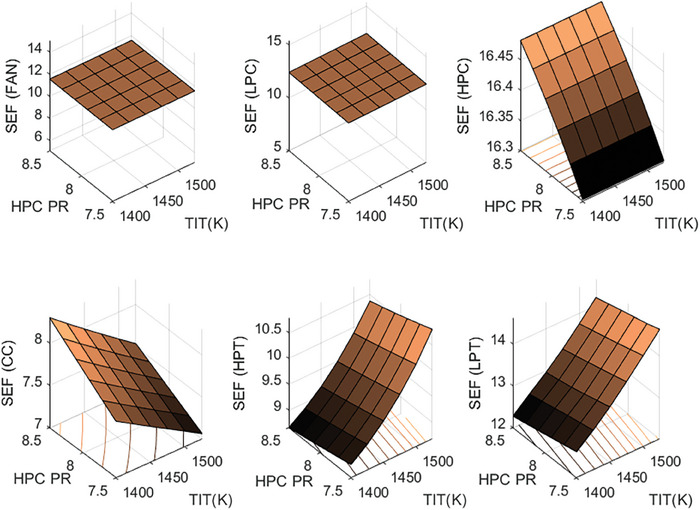
Sustainable efficiency factor of the HBP‐TF engine components at different cases.

**Figure 15:**
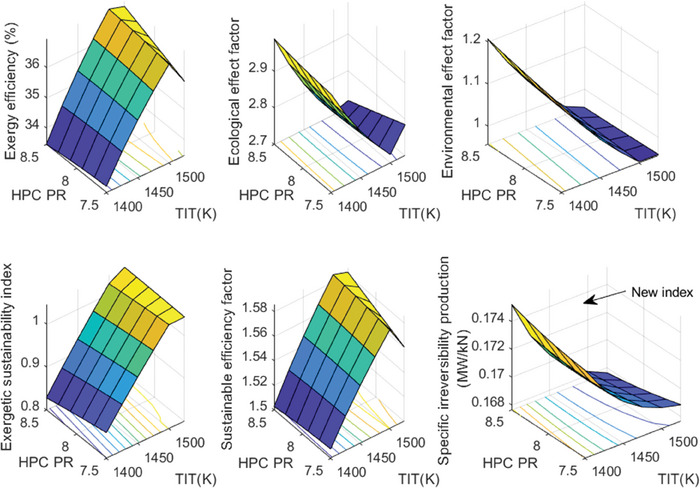
Thermodynamic indicators of the overall HBP‐TF engine at different cases.

This section covers evaluations of entropy, exergy, and environmental and sustainability parameters at both baseline and several points, which consist of different TIT and HPC‐PR values. To compute these parameters, the engine is subjected to thermodynamic and parametric cycle analyses in MATLAB environment. Thus, the effects of each design variable on engine‐specific metrics could be observed with graphics. For clarity, this section could be divided into three subsections. Entropy findings are presented in **Figures** **3** and **4**, component‐based exergetic outcomes for six components are given in **Figures** 5, 6, 7, 8, 9, 10 and environmental and sustainability parameters are shown in **Figures** 11, 12, 13, 14, Lastly, **Figure** **15** involves the thermodynamic indicators of the overall engine. Before discussion, note that while considering effect of HPC PR, TIT is kept constant as 1450 K, whereas HPC PR is considered as 8.1 during the investigation of the effect of TIT.

Figure 3 incorporates several entropic metrics such as specific entropy, entropy production, isentropic efficiency, and entropic improvement potential (EIP) at baseline. The specific entropy increases from the Fan outlet to the LPT outlet. The highest specific entropy increment occurs in the combustor with 0.74 kJ kgK^−1^. In this regard, the combustor has a significant entropy production of 0.044 MW K^−1^. For turbomachinery components, the isentropic efficiency is computed. The lowest efficiency is observed at HPT with 79.16% whereas the highest efficiency is determined at Fan with 90.05%. As for EIP, the highest EIP belongs to LPT with 0.80 kW K^−1^ while the lowest one occurs in LPC with 0.19 kW K^−1^.

Figure 4 demonstrates that entropy production occurred in the components throughout the different cases. As can be seen, except combustor, the higher HPC PR leads to increase entropy production, whereas it holds vice versa for the effect of TIT. The entropy production of Fan and LPC has no variation due to alterations in design parameters. Their values are 4.9 and 1.6 kW K^−1^ for Fan and LPC, respectively. Moreover, when considering the effects of HPC‐PR and TIT for the other components, entropy production of the combustor decreases from 46.09 to 43.4 kW K^−1^ whereas entropy production of HPC, HPT, and LPT increases from 5.5 to 5.9 kW K^−1^, from 2.5 to 2.8 kW K^−1^ and from 4 to 4.2 kW K^−1^ due to rising HPC‐PR, respectively. However, the effect of TIT is observed on several components except for Fan, LPC, and HPC. The combustor has varying entropy production from 41.59 to 48.48 kW K^−1^, whereas the entropy production of HPT and LPT diminishes from 2.92 to 2.59 kW K^−1^ and from 4.64 to 3.90 kW K^−1^, respectively with rising turbine inlet temperature. Concisely, entropy production of the combustor ascends by 16.56% due to increased TIT and by 5.83% with an increase in HPC‐PR, which means that the effect of TIT is more discernable than HPC‐PR impact.

On the other hand, the results of exergetic indicators involving exergy destruction, exergy efficiency, waste exergy ratio, fuel exergy waste ratio, improvement potential rate, and improved exergy are presented for the baseline and different cases. Before analyzing the design effects on exergetic metrics, **Table** **1** presents the parameters related to exergetic assessments at baseline. These outcomes could help in comparing the effects of HPC‐PR and TIT on the exergetic performance of the HBP‐TF engine. According to Table 1, exergy destruction of the engine is estimated as 18.83 MW whereas exergy efficiency and improved exergy efficiency are measured as 35.41% and 46.83%, respectively at baseline.

**Table 1:** Exergetic indicators of the HBP‐TF engine under baseline.

Components	ExD [MW]	***ψ*** [%]	WExR [%]	FExWR [%]	Ex_IP [MW]	Ψ [%]
FAN	1.42	91.34	4.41	2.85	0.12	92.03
LPC	0.48	91.94	1.51	1.02	0.03	92.54
HPC	1.52	93.91	4.73	3.06	0.09	94.26
CC	10.52	87.11	32.58	21.03	1.35	88.59
HPT	2.98	89.22	9.24	5.96	0.32	90.27
LPT	1.88	92.35	5.84	3.77	0.14	92.90
Turbofan engine	18.83	35.41	58.31	37.64	2.07	46.83

First, the exergy efficiencies of the components are evaluated for different cases in Figure 5. The exergy efficiency of Fan and LPC is constant throughout all cases, whereas that of HPC is affected only by variation of HPC‐PR. However, those of the combustor, HPT, and LPT, are affected by both variables. In this regard, Fan and LPC have exergy efficiency of 91.34% and 91.94%, respectively while that of HPC is observed between 93.87% and 93.93% due to rising HPC‐PR. However, exergy efficiencies of the combustor, HPT, and LPT vary from 86.32% to 87.23% (increment), 89.83% to 89.47% (decrement), and 92.73% to 92.5% (decrement) owing to increasing HPC‐PR. As the influence of TIT, exergy efficiencies of these three components change from 87.57% to 86.39% (decrement), 88.59% to 90.5% (increment), and 91.94% to 93.04% (increment) due to rising TIT.

As another indicator, the variation of exergy destruction for six components is presented in Figure 6. The exergy destruction (Ex_dest_) of Fan and LPC were found to be 1.42 and 0.48 MW, respectively. These are constant for each case. However, the Exdest of the combustor is measured as 10.52 MW at baseline, whereas it decreases from 11.01 to 10.21 MW, which means 7.26% of decrement due to increased HPC‐PR, whereas it raises from 9.61 to 12.01 MW, which corresponds 24.97% increment owing to the elevated TIT. Alternatively, Ex_dest_ of HPC, HPT, and LPT increase by 7.27%, 11.87%, and 3.26%, respectively, as HPC‐PR increases, whereas those of HPT and LPT decrease by 18.55% and 14.57%, respectively due to raising TIT. It can be underlined that exergy destruction is more sensitive against to increment of TIT. Also, these findings agree well with the results of entropy production of the components.

Figure 7 shows the wasted exergy ratio (WExR) of the six components of the HBP‐TF engine. This index shows how much the irreversibility occurs in the component per irreversibility of the overall system. It could be stressed that the WExR of each component is affected by variations in HPC‐PR and TIT. The reason for this is that wasted exergy is prone to change with alterations in the design variables. Furthermore, except for the combustor, the WExRs of the other five components decrease because of increasing TIT. In this context, WExR of the combustor is measured as 32.58% at baseline, whereas it increases by 1.72% with effects of the higher TIT and decreases by 1.05% due to raising HPC‐PR. Furthermore, the higher TIT leads WExR of Fan, LPC, HPC, HPT, and LPT to diminish by 0.74%, 0.25%, 0.79%, 3.28%, and 1.85%, respectively. On the other hand, as HPC‐PR increases, WExRs increase by 0.19% at Fan, 0.06% at LPC, 0.53% at HPC, 1.43% at HPT, and 0.43% at LPT. According to these results, the combustor produces the highest irreversibility, unfavorably affecting sustainability.

Figure 8 demonstrates how the fuel‐exergy waste ratio changes with the alteration of design variables throughout different cases. This parameter determines wasted exergy that emerged in each component per unit of fuel exergy for different cases. Among all components, the FExWR of the combustor is observed to be the highest. Increasing TIT assists in dropping FExWR for all components except the combustor. This case does not hold for the rising of HPC‐PR. Accordingly, the FExWR of the combustor is measured as 21.03% at baseline, whereas it increases by 0.19% with effects of the higher TIT and decreases by 0.69% due to raising HPC‐PR. Furthermore, the higher TIT leads FExWR of Fan, LPC, HPC, HPT, and LPT to decrease by 0.6%, 0.2%, 0.64%, 2.38% and 1.36%, respectively. Moreover, with increasing HPC‐PR, the FExWRs increase by 0.12% at Fan, 0.04% at LPC, 0.34% at HPC, 0.92% at HPT, and 0.28% at LPT. This means that the variation of HPC‐PR affects the FexWR by less than 1% in the considered ranges.

Figure 9 depicts the exergetic improvement potential (ExIP) for each component throughout the different cases. This indicator identifies how much improvement occurs in the components in case of the irreversibility removed from the components. As expected, the alteration of HPC‐PR and TIT does not affect ExIP of the Fan and LPC. Their values are estimated at 0.12 and 0.03 MW, respectively. The ExIP of the combustor is computed as 1.356 MW at baseline, whereas it ascends by 36.92% with the impact of the higher TIT and diminishes by 13.75% due to increased HPC‐PR. However, ExIP of HPC, HPT, and LPT increased by 6.12%, 15.9%, and 6.2%, respectively, as HPC‐PR increased, whereas those of HPT and LPT decreased by 32.18% and 26.19%, respectively due to raising TIT.

The last exergetic metric is the improved exergy efficiency (IMPExEF) shown in Figure 10. This parameter reflects the effect of the exergetic improvement rate on the exergy efficiency. In this context, IMPExEF of the Fan and LPC is calculated as 92.03% and 92.54%. These are constant throughout all cases. However, the IMPExEF of the combustor is measured as 88.59% at baseline, whereas it enhances from 88.13% to 88.87%, which means 0.44% of increment due to increased HPC‐PR, whereas it deteriorates from 88.95% to 88.02%, which corresponds 0.93% decrement owing to the elevated TIT. However, IMPExEF of HPC increases by 0.06% whereas those of HPT and LPT decrease by 0.3% and 0.19%, respectively as HPC‐PR increases. Also, the IMPExEF of HPT and LPT enhances by 1.57% and 0.95%, respectively due to raising TIT. It could be deduced that improved exergy of components is affected by TIT more compared with the HPC‐PR effect.

Indicators regarding sustainability and environment are evaluated for the HBP‐TF engine components. For these assessments, there are four parameters consisting of the ecological effect factor, environmental effect factor, exergetic sustainability index, and sustainable efficiency factor. These metrics are computed at the baseline presented in **Table** **2**. These are important to observe the effects of design variables on the sustainability and environmental performance of the engine. Note that the four parameters mentioned are non‐dimensional since they are obtained by dividing exergetic relations.

**Table 2:** Environmental and sustainability metrics of the engine under baseline.

	Environmental metrics		Sustainability metrics	
Components	EcoEF	EEF	ExSI	SEF
FAN	1.09	0.03	32.06	11.54
LPC	1.08	0.01	94.49	12.40
HPC	1.06	0.03	30.73	16.41
CC	1.14	0.24	4.142	7.76
HPT	1.12	0.06	14.96	9.27
LPT	1.08	0.04	24.48	13.07
Turbofan engine	2.82	1.06	0.94	1.54

Figure 11 presents the ecological effect factor of six components for different cases. This index has an adverse relationship with exergy efficiency. Increasing the exergy efficiency leads to lowering EcoEF. In this sense, the highest EcoEF belongs to the combustor, whose value is measured as 1.1479 at baseline. The EcoEF of the combustor tends to ascend from 1.1419 to 1.1575, which expresses 1.36% of increment owing to elevated TIT. However, it decreases from 1.1556 to 1.1432, which corresponds to 1.07% decrement due to raising HPC‐PR. As for EcoEF of the Fan and LPC, these are estimated as constant with 1.094 and 1.087, respectively. Alternatively, with the higher TIT effect, EcoEFF of HPT and LPT decrease by 2.11% and 1.17%, respectively. Moreover, these metrics increase by 0.402% and 0.231% due to the elevated HPC PR. Lastly, EcoEF of HPC is affected only from the effect of HPC‐PR, which decreases by 0.065%.

Figure 12 shows the variation of environmental effect factor (EEF) of the HBP‐TF engine components versus alterations of design variables. This index is associated with exergy efficiency and wasted exergy ratio. Namely, the higher exergy efficiency and the lower wasted exergy ratio result in a lower EEF. In this context, the highest EEF belongs to the combustor, whose value is computed as 0.2414. As the TIT increases, EEF of the combustor increases from 0.2401 to 0.2455, which means 2.24% increment whereas it diminishes from 0.2479 to 0.2373, which corresponds to 4.24% decrement. The EEFs of Fan, LPC, HPC, HPT, and LPT decrease by 19.06%, 19.13%, 19.38%, 35.62%, and 31.93% owing to the higher TIT whereas those increase 4.27%, 4.85%, 11.84%, 17.52%, and 7.96% due to raising HPC‐PR.

Figure 13 demonstrates the exergetic sustainability index of the components for different cases. This index is related to the environmental effect factor. The higher the EEF, the lower the ExSI. In this regard, the highest ExSI belongs to LPC, which is measured to be 94.49 at baseline, whereas, as expected, the combustor enjoys the lowest ExSI that is 4.1423. Increasing TIT makes the ExSI of the combustor lower, which changes by 2.21% whereas raising HPC‐PR leads it to increase by 4.44%. However, with increments of TIT, ExSI of Fan, LPC, HPC, HPT, and LPT ascend by 23.92%, 23.92%, 23.93%, 55.42%, and 46.81%, respectively while ExEFFs diminish by 4.16% at Fan, 4.15% at LPC, 10.6% at HPC, 14.83% at HPT, and 7.28% at LPT owing to the increased HPC PR.

The sustainable efficiency factor of the HBP‐TF engine components is presented in Figure 14. This indicator has a positive relationship with the exergy efficiency. This means that to obtain a higher SEF, exergy efficiency should be higher. According to this, the combustor has the lowest SEF, which is computed as 7.76. The higher TIT leads SEF of the combustor to decrease by 8.7%, whereas the higher HPC‐PR ends with an increment by 7.51%. When considering the other components, as the TIT becomes the higher, SEFs of Fan, LPC, and HPC are constant as 11.54, 12.4, and 16.41, respectively. Moreover, SEFs of HPT and LPT increase by 20.1% and 15.71%, respectively. Furthermore, as HPC‐PR reaches the higher, SEF of HPC increases by 1.09% whereas, SEF of HPT and LPT diminishes by 3.28% and 2.85%, respectively.

Considering the assessments made between Figures 5, 6, 7, 8, 9, 10, 11, 12, 13, 14, it could be deduced that HPC‐PR and TIT do not have the same effect on the exergetic and sustainability‐based parameters of the six components. Therefore, it can be useful to evaluate these metrics for the overall engine.

In this framework, six different indicators for the HBP‐TF engine are presented in Figure 15. According to this figure, the exergy efficiency (ExEF) of the engine is observed as 35.41% under baseline, whereas it is affected favorably up to 1500 K of TIT. Namely, it increases from 33.61% to 36.83%. After this point, it decreases to 36.26%. On the other hand, the effect of HPC‐PR on ExEF is not as discernible as the TIT effect. Accordingly, the ExEFF of the engine increases from 35.36% to 35.40% due to the increased HPC‐PR. However, the ecological effect factor of the engine is measured as 2.8240 at baseline. Similarly, it diminishes from 2.9749 to 2.7580 due to the elevated TIT whereas the engine's EcoEF tends to slightly decreases from 2.8278 to 2.8249. Moreover, the environmental effect factor of the HBP‐TF engine is computed as 1.0630 at baseline. With the higher TIT, the EEF of the engine decreases from 1.1880 to 0.9572 while increasing HPC PR, it from 1.0476 to 1.0749. Additionally, the exergetic sustainability index of the overall engine is determined as 0.9407 under baseline. The higher TIT favorably affects the ExSI of the engine, which increases from 0.8417 to 1.0448 with an increment in TIT. However, as the HPC PR reaches its highest value, the ExSI of the engine drops from 0.9546 to 0.9303. Also, the sustainable efficiency factor of the engine is 1.5482 at baseline. Increasing TIT leads the SEF to raise from 1.5054 to 1.5732 whereas increasing HPC PR slightly increases it from 1.5471 to 1.5480. Lastly, as a new index, specific irreversibility production of the overall engine is computed as 0.1705 MW kN^−1^ at baseline. When considering the TIT effect, it causes the SIP to lower from 0.1746 to 0.1680 MW kN^−1^. This finding agrees will the results of other indicators regarding the engine. As for the HPC PR effect, it leads to SIP to slightly decrease from 0.1707 to 0.1705 MW kN^−1^. Based on the six parameters shown in this figure, it could be deduced that these metrics help us determine optimum design values to obtain the highest sustainability value.

## 5 Conclusion

Evaluating the performance behavior of the kerosene‐fueled high by‐pass turbofan (HBP‐TF) engine with significant design variables is of high importance  for assessing the gas turbine engine. In this study, the pressure ratio of the high‐pressure compressor (HPC‐PR) varying from 7.5 to 8.5 and turbine inlet temperature (TIT) ranging from 1400 to 1525 K are chosen to observe the effects of these variables on several exergetic and environmental indicators for different cases that reflect variable needs in the aviation industry. To compute these parameters, the engine is subjected to thermodynamic and parametric cycle analyses in a MATLAB environment. Moreover, to facilitate evaluations regarding exergetic metrics, the baseline values of these indicators are presented. The thermodynamic analysis performed for the main components is extended to the overall turbofan engine. As a novelty, entropic improvement potential (kW K^−1^) is measured for turbomachinery components and the specific irreversibility production (MW kN^−1^) of the overall engine is first measured for this engine in different cases. **Table** **3** shows comparative findings of previous studies with the current one. It is shown that these results are consistent with the present study.

**Table 3:** Comparison of the findings of turbofan engine.

Researchers	Year	Engine	Applied analyses	Application point	Exergy efficiency
Aydin et al.^[^ ^37^ ^]^	2015	CFM56‐7B	Exergy	One point (Take‐off)	31.5%
Korba et al.^[^ ^38^ ^]^	2023	CFM56‐3	Exergy and exergo‐economic	One point (Take‐off)	33.32%
Present study	2023	CFM56‐7B	Entropy and exergy	Thirty‐six points (Different TIT and HPC PR values)	33.61%−36.83%

The main outcomes obtained from thermodynamic evaluations could be presented as follows:

- Among all the main components of the engine, the combustor is the main source of irreversibility, which deteriorates the sustainability of the engine. Specifically, the entropy production in the combustor is calculated as 44.4425 kW K^−1^. The higher TIT increases the entropy production of the combustor by 16.56% whereas it diminishes by 5.83% with the higher HPC PR.
- When considering the effects of HPC PR and TIT, since each component is affected to a different extent, determining the lowest entropy production requires finding optimum design points on a component basis.
- The exergy efficiency of the combustor was found to be 87.11% at baseline. Increasing HPC‐PR leads to an increase in exergy efficiency of the combustor, which rises by 0.94%, whereas an increment in TIT lowers this metric by 1.17%.
- The wasted exergy ratio of the combustor was determined to be 32.58% at baseline. Increasing TIT and HPC PR causes WExR to increase by 1.82% and to decrease by 1.05%, respectively. Except for the combustor, the WExR of other components has a similar tendency in response to the effect of variables.
- The improved exergy efficiency of the components is computed as higher than their exergy efficiency. Accordingly, the improved exergy efficiency of the combustor is measured as 88.59% at baseline. The improvement in ExEFF of the combustor is 1.48%. Moreover, the enhancement in ExEFF for the Fan, LPC, HPC, HPT, and LPT is 0.69%, 0.60%, 0.35%, 1.05%, and 0.55%, respectively.
- The exergetic sustainability index of the combustor is quantified as 4.1423 at baseline. It becomes lower, which decreases by 2.21% as TIT increases. However, the higher HPC‐PR leads to an increase in the ExSI of the combustor by 4.49%.
- The exergy efficiency of the overall engine is computed as 35.41% at baseline, which increases by 2.86% due to increased TIT and by 0.04% due to elevated HPC‐PR. Namely, the effect of TIT is more discernible than the HPC‐PR effect.
- The sustainable efficiency factor of the engine is favorably affected by both the higher TIT and HPC‐PR. SEF of the engine is measured as 1.5482 at baseline whereas it increases by 4.5% and 0.058% with an increment of TIT and HPC‐PR, respectively.
- Finally, the finding of the specific irreversibility production of the overall engine agrees well with the results of ExEFF and SEF. The higher TIT and the higher HPC‐PR result in an improvement of the engine sustainability. Namely, SIP of the engine decreases by 3.78% and 0.1171% as the TIT and HPC PR increase up to the highest value considered in the study.

Considering the findings discussed above, an investigation on the effects of design variables helps to determine whether the engine has potential for a more sustainable run. Accordingly, the component‐based sustainability computations that cover the overall engine provide a clearer perspective on the effect of design parameters on engine sustainability. It is thought that this study offers significant outcomes for finding optimum design variables by detecting the potential saving points with less environmental impact. Namely, coupling thermodynamic laws with gas turbine engines assists in understanding optimum engine parameters for sustainability. Considering entropy, exergy, and environmental metrics together for different cases is of high importance to determine which case leads to lower environmental damage in terms of thermodynamics. For the next study, multi‐objective optimization of entropy‐based and exergy‐based metrics with metaheuristic methods could be performed Moreover, some thermodynamic parameters of the engine with deep learning approaches could be predicted by considering flight and design conditions.

## Conflict of Interest

The authors declare no conflict of interest.

## Data Availability

The data that support the findings of this study are available from the corresponding author upon reasonable request.
